# Vital Statistics of Christian County, Ky.

**Published:** 1852-05

**Authors:** W. A. Edmonds

**Affiliations:** Hopkinsville, Ky.


### WESTERN JOURNAL

OF

### MEDICINE AND SURGERY.

MAY, 1852.

Art. I.—*Vital Statistics of Christian County, Ky.* By W. A. Edmonds, M. D., of Hopkinsville, Ky.

The county of Christian originally embraced a large scope of country in the south-western part of Kentucky, which has since been divided and subdivided into a number of new counties. The first settlement within the present limits of the county was made in the neighborhood of Hopkinsville, in the year 1792 or 1793. Christian ranks among the first counties in the southern part of the State, in point of population and for the intelligence and moral respectability of its inhabitants, as well as for its natural and especially its agricultural resources. The county is naturally divided into a northern and southern portion by a line running from east to west through its entire width. The face of the country in the southern part is mostly very level, but in places is gently undulating. The soil is a deep black, rich loam, smartly interspersed with sand, lying on a foundation of deep red clay, with a substratum of mountain lime-stone at an average of about 12 feet from the surface. This portion of the county is favorable to the production of Indian corn, tobacco, rye, wheat, oats, and clover; but little has been done in the culture of hemp. This part of the county is naturally not well watered, owing to the cavernous character of the lime-stone; but the enterprise of our farmers in the formation of ponds, and digging wells and cisterns, has done much to remedy this deficiency.

The northern part of the county is less adapted to agriculture, being broken and uneven, and in places is characterized by steep bluffs and rugged ledges of lime-stone; but it abounds in magnificent forests, stone-coal, game, fish and fruit. It is abundantly provided with excellent water by the various springs that issue from its bluffs and hill-sides.

The primitive log cabin, with its wooden chimney, is rapidly giving way to the more commodious and tasteful mansion of brick or frame-work. Much attention is being given to education and the dissemination of christianity. Almost every neighborhood has its church and school-house. The value of church property by the census was $54,200, and the churches it was computed would accommodate 12,275 persons. There are three newspapers in the county, all published at Hopkinsville, and well sustained. There are several well-selected and respectable private libraries in the county, but I have no means of ascertaining the number of volumes.

The county has 10 divisions of the Sons of Temperance and a temple of honor, with an aggregate of 436 paying members. The Odd Fellows have 2 lodges and about 100 members. The Free Masons number 191.

The second Kentucky Lunatic Asylum is located within two miles of Hopkinsville, on the Lexington road, and is designed to accommodate, when completed, 300 inmates. Its architectural style and arrangement are both in the best taste and highly judicious; and when finished the edifice will be the most splendid in the State. It will constitute a lasting monument to the enlightened benevolence of the State, in behalf of a most afflicted and unfortunate class of her citizens. It was commenced in 1848, and is expected to be ready for a small number of patients by the end of the present year.

The county is provided with a public Poor-house for the accommodation of paupers; but such is the happy and prosperous condition of our population, that application for such assistance is seldom made, and when made is usually on account of great age or serious afflictions. According to the last census there were but six paupers in the county.

Disease in an endemic or sporadic form frequently prevails, but the county has rarely been visited by an epidemic. Sporadic cases of cholera in hot weather sometimes occur, but no epidemic of that disease has ever visited the county.

Hopkinsville, the seat of justice, is located near the middle of the county, in a healthy and fertile region, and at a point to command all its peculiar productions and resources. It has a city charter with the usual police and corporate regulations and privileges. It has many excellent private residences, a neat and tasteful Court-house, and in the number and elegance of its edifices for public worship, is probably not excelled by any town of its size in the west.

By the census of 1850 there were in the county

| | |
|---|---|
| White inhabitants, - - - | 11,462 |
| Slaves, - - - - - | 8,238 |
| Total, - - - - - | 19,700 |

The estimate of white inhabitants includes the few free negroes in the county—there being no convenient mode in the arrangement of the census report for making the distinction. The white females were in proportion of $18\frac{13}{20}$ to $21\frac{7}{20}$ males. The females in the black population were in the proportion of 22 to 18 males. The difference in ratio of the sex among the two races is probably owing to the exportation of negro men to the southern states, rather than to any relative difference of longevity in the two races. The average age of the whites was 21-89 years; that of the slaves 18.63 years.

The following is a table of marriages for the last six years, up to 1st January, 1852:

TABLE OF MARRIAGES.

| | 1846. | 1847. | 1848. | 1849. | 1850. | 1851. | AVERAGE. |
|---|---|---|---|---|---|---|---|
| January, | 10 | 10 | 14 | 13 | 17 | 17 | 13.50 |
| February, | 7 | 11 | 13 | 10 | 4 | 6 | 8.50 |
| March, | 6 | 9 | 10 | 5 | 9 | 5 | 7.33 |
| April, | 3 | 4 | 3 | 5 | 5 | 2 | 8.66 |
| May, | 4 | 3 | 2 | 5 | 4 | 3 | 3.5 |
| June, | 8 | 8 | 5 | 7 | 6 | 2 | 6. |
| July, | 7 | 3 | 1 | 9 | 9 | 9 | 6.33 |
| August, | 5 | 7 | 6 | 5 | 3 | 9 | 5.83 |
| September, | 11 | 11 | 8 | 9 | 4 | 4 | 7.83 |
| October, | 11 | 7 | 14 | 10 | 5 | 5 | 7. |
| November, | 13 | 8 | 10 | 7 | 4 | 10 | 8.66 |
| December, | 12 | 11 | 6 | 13 | 14 | 11 | 12.83 |
| Total, | 97 | 92 | 92 | 98 | 84 | 83 | |

The deaths for the year ending 1st January, 1850, were 970—135 males and 136 females; 143 of the number were slaves. The following table shows the mortality for each month during that year:

| 1850. | DEATHS. | 1850. | DEATHS |
|---|---|---|---|
| January, | 29 | July, | 23 |
| February, | 22 | August, | 33 |
| March, | 29 | September, | 24 |
| April, | 24 | October, | 17 |
| May, | 23 | November, | 12 |
| June, | 18 | December, | 14 |
| | 145 | | 123 |
| | | | 145 |
| Total, | | | 270 |

The average age at which death occurred, was 12.84 years. The following is an exhibit of mortality for each decade of life:

| | | | | | |
|---|---|---|---|---|---|
| Under | 1 year of age, | | | | 56 |
| Over | 1 and under | 10 years, | | | 65 |
| " | 10 | " | 20 | " | 38 |
| " | 20 | " | 30 | " | 33 |
| " | 30 | " | 40 | " | 23 |
| " | 40 | " | 50 | " | 17 |
| " | 50 | " | 60 | " | 10 |
| " | 60 | " | 70 | " | 17 |
| " | 70 | " | 80 | " | 7 |
| " | 80 | " | 90 | " | 2 |
| " | 90 | " | 100 | " | 0 |
| " | 100 | " | 110 | " | 0 |
| " | 110 | " | 120 | " | 0 |
| " | 120 | " | 130 | " | 1 |
| Total, | | | | | 270 |

The greatest age attained by any individual appears from the census to have been 125 years; and this was a negro woman.

From my personal recollections and my examination of the census, I am induced to believe that but little confidence is to be placed in the census reports as to the character of disease from which the deaths reported took place. This remark is not intended to reflect upon the gentleman who took the census, but the fact is attributed to the negligence or incompetency of those who reported the deaths and their causes. According to the report, of the 270 deaths, 55 were from typhoid fever; 19 from consumption; 11 from croup; 9 from pneumonia; and 46 from causes unknown. The remainder were about equally divided between the various affections which are common to every portion of the state. The average duration of the typhoid fever cases, was 18 days. This disease has been the prevailing disease of the country for the last few years, and seems to have taken the place of our intermittent and remittent forms of fever. It is confined to no particular month, locality, sex, or condition, or period of life, except that infancy seems to be exempt from the disease. It is usually complicated with affections of the lungs, bowels, or brain; but is rarely attended with any eruptive appearances.

There are 33 practitioners of medicine in the county. Of these, 25 practice as regular physicians; the other 8 are Homœopathists, Steamers, Eclectics, Indian doctors, &c. There is no medical society in the county. The rate of charges agreed upon by the faculty is fair and remunerative, and, in the main, is adhered to. It is held to be unprofessional to attend families by the year, and I believe that, with a few individual exceptions, the obligation not to do so is scrupulously maintained.

Hopkinsville, Ky., March 30th, 1852.

